# Rapidly Boosted Plasma IL-5 Induced by Treatment of Human *Schistosomiasis haematobium* Is Dependent on Antigen Dose, IgE and Eosinophils

**DOI:** 10.1371/journal.pntd.0002149

**Published:** 2013-03-28

**Authors:** Shona Wilson, Frances M. Jones, Hassan K. M. Fofana, Aissata Doucouré, Aly Landouré, Gachuhi Kimani, Joseph K. Mwatha, Moussa Sacko, Birgitte J. Vennervald, David W. Dunne

**Affiliations:** 1 Department of Pathology, University of Cambridge, Cambridge, United Kingdom; 2 Institut National de Recherche en Santé Publique, Bamako, Mali; 3 Kenya Medical Research Institute, Nairobi, Kenya; 4 DBL – Centre for Health Research and Development, Faculty of Life Sciences, University of Copenhagen, Frederiksberg C, Denmark; René Rachou Research Center, Brazil

## Abstract

**Background:**

IgE specific to worm antigen (SWA) and pre-treatment eosinophil number, are associated with human immunity to re-infection with schistosomes after chemotherapeutic treatment. Treatment significantly elevates circulating IL-5 24-hr post-treatment of *Schistosoma mansoni*. Here we investigate if praziquantel treatment of human schistosomiasis haematobium also boosts circulating IL-5, the immunological and parasitological factors that predispose to this, and the relationship between these and subsequent immunity to post-treatment re-infection.

**Methodology/Principle Findings:**

The relationship between pre-treatment SWA-IgE, eosinophil number and infection intensity and the 24-hr post-treatment IL-5 boost was investigated in a Malian cohort (aged 5–40 yrs), exposed to *S. haematobium*. Eotaxin levels were measured at 24-hr post-treatment as a proxy of eosinophil migration. The relationship between the 24-hr post-treatment IL-5 boost and later eosinophil numbers and SWA-IgE levels (9-wk post-treatment) was examined, then investigated in the context of subsequent levels of re-infection (2-yr post-treatment). Circulating IL-5 levels increased 24-hr post-treatment and were associated with pre-treatment infection intensity, SWA-IgE levels, eosinophil number, as well as 24-hr post-treatment eotaxin levels. 24-hr IL-5 levels were, in turn, significantly associated with eosinophil number and elevated SWA-IgE 9-wk later. These SWA-IgE levels were significantly associated with immunity to re-infection.

**Conclusions/Significance:**

Early IL-5 production after treatment-induced exposure to *S. haematobium* worm antigen is positively associated with antigen dose (infection intensity), IgE availability for arming of effector cells at time of treatment and subsequent eosinophil migration response (as indicated by eotaxin levels). The IL-5 produced is positively associated with increased downstream eosinophil number and increases in specific IgE levels, implicating this cytokine boost and its down-stream consequences in the production and maintenance of IgE, and subsequent re-infection immunity.

## Author Summary

Partial human immunity to infection with trematode worms of the genus *Schistosoma* is associated with IgE specific to adult worm-derived antigens and eosinophils. Treatment studies of *Schistosoma* infection allow us to examine the temporal features of the immune response post-antigen exposure, their inter-dependence and their relationship with re-infection levels. Here the boosted levels of the cytokine IL-5, measured at 24-hrs post-treatment of a Malian cohort, aged 5–40 yrs, were found to be significantly associated with pre-treatment levels of IgE to worm-derived antigens and eosinophil number, linking this rapid response to two of the main correlates of human immunity to these parasites. The IL-5 levels at 24-hr were in turn related to increased eosinophil counts and SWA-IgE levels at 9-wks post-treatment. In line with previous studies SWA-IgE was associated with resistance to re-infection. The study therefore identifies temporal relationships between immune mediators prior to and post treatment induced antigen exposure that are associated with resistance to re-infection.

## Introduction

*Schistosoma haematobium* infection is the most prevalent form of human schistosomiasis and a major public health problem. Schistosomiasis haematobium is associated with bladder calcification, hydronephrosis, kidney failure, lesions of the genital tract and bladder carcinoma. *S. haematobium* infection intensities follow a similar age distribution to those of other human schistosome infections – rising throughout childhood, peaking in early adolescence, before declining sharply in late adolescence and early adulthood. Epidemiological studies of *S. mansoni* infection in fishing communities, where exposure to infection is greatest in adult males, show that this characteristic age-infection intensity curve is still present, and thus maintained by factors other than exposure to infection [1]. This implies that in endemic communities, a partial immunity to re-infection slowly develops, and that this significantly contributes to the decline in infection intensities seen in older members of schistosomiasis endemic communities.

Immuno-epidemiological studies that use a treatment/re-infection study design - in which immune mediators are measured, a cohort treated and their re-infection levels determined 1 to 2-yr later - have shown IgE levels, specific for schistosome adult worm derived antigens (SWA), to be a major correlate of immunity to infection with each of the 3 schistosome species that cause most human schistosomiasis [2], [3], [4]. Pre-treatment circulating eosinophil number is also associated with immunity to re-infection with both *S. mansoni* [5], [6] and *S. haematobium* [7]. In schistosomiasis endemic areas, human SWA-specific IgE levels increase with age [8], [9], [10], and in response to praziquantel treatment [3], [11], [12], [13]. Adult schistosome worms live for many years in the human blood stream, but treatment disrupts the integrity of the worm's outer tegument exposing a range of formerly cryptic antigens to the host's immune system [14]. The post-treatment IgE levels induced by otherwise cryptic antigens can be more strongly associated with re-infection immunity than pre-treatment IgE levels [3], [13], and multiple rounds of treatment is found to increase resistance [15]. Eosinophil number also alters dramatically post-treatment, peaking between 2 and 4-wk post-treatment [16], [17].

High levels of IL-5 are produced by PBMC and whole blood cell cultures stimulated in vitro with SWA [18], [19], [20], [21] and pre-treatment in vitro SWA specific IL-5 responses are associated with post-treatment SWA-IgE levels [22], suggesting that IL-5 responsiveness is a key component of human immunity to schistosomiasis. In adult fishermen infected with *S. mansoni*, plasma IL-5 is elevated 24-hr post treatment [17]. Human IL-5 production is therefore dynamic in response to antigen exposed by chemotherapeutic treatment for this parasite. Circulating eosinophil number declines concurrently with the increased plasma IL-5 levels 24-hour post-treatment, suggesting that these cells migrate to tissues responding to antigen released by treatment, and that they may play a role in the production of the elevated IL-5 [17].

We conducted a re-infection study in a *S. haematobium* endemic Malian population, to determine whether an equivalent to the boost in IL-5 that occurs after treatment of *S. mansoni* occurs after treatment for *S. haematobium* infections, and to model any such IL-5 boost against known key components of immunity to schistosomiasis, SWA-IgE and eosinophils. The interdependence of IL-5, SWA-IgE and eosinophils was then modeled in relation to subsequent immunity to re-infection by the parasite. IL-5 levels were boosted 24-hr post-treatment and found to be dependent on pre-treatment SWA-IgE levels, eosinophil count and infection intensity. IL-5 levels 24-hr post-treatment were also significantly associated with increased SWA-IgE levels 9-wk post-treatment, which in turn were associated with partial immunity to re-infection.

## Materials and Methods

### Ethics Statement

The study received ethical approval from the Ethical Review Committee of the National Institute for Research in Public Health, Mali. Informed oral consent was given by the chief of the villages and adults during village meetings. Informed oral consent was given on an individual basis by adult participants and for children by their parents or guardians during recruitment. Consent was recorded by the recruitment team. Due to cultural reasons and low literacy rates in villages, oral consent is deemed acceptable by the Malian Ministry of Health and was approved by the Ethical Review Committee of the National Institute for Research in Public Health.

### Study Villages and Cohort

The study took place in 3 villages, Segou Region, Mali. Two villages, Kaladangan and Guenidaga, are fishing settlements on the banks of the River Niger. The third village, Kalabougou, is on a tributary of the main river and occupation is more diverse, consisting of fishing, farming and pottery. The cohort of 326 individuals, 186 females and 140 males, aged 5 to 40 yrs, were selected from the village populations by generation of random numbers, with the sole selection criteria being aged between 5 and 40 years of age. A number of school-aged children were excluded from the selection as they had previously been treated by the Schistosome Control Initiative. The cohort were part of a multi-disciplinary study to assess the impact of one v two treatment and were assigned by random selection, to receive either a single dose of praziquantel, or a second dose 2-wk after the first. At 9-wk post-treatment 245 individuals participated and 167 who participated in all previous time-points were followed-up 2-yr later. Three urine samples were collected from each individual pre-treatment and 10 ml of each was filtered for *S. haematobium* egg counts. Three urine samples were collected 9-wk post-treatment to assess efficacy of treatment, and three samples were collected 2-yr post-treatment to assess re-infection intensities. Pre-treatment stool samples were examined for *S. mansoni* and gut nematode infections by the Kato Katz method.

### Plasma Preparation

Five ml of blood were collected by venipuncture into EDTA, pre-treatment and 9-wk post-treatment. A 200 µl aliquot of whole blood was removed for haematology analysis and preparation of May-Grünswald stained blood smears for eosinophil differential counts. Eosinophil number/ml of blood was calculated from the differentials and total white blood cell counts. After centrifugation of remaining blood samples the plasma was harvested. Finger-prick blood samples, collected into EDTA, were taken pre-treatment and 24-hr post-treatment, centrifuged and the plasma harvested. Plasma samples were stored at −20°C prior to shipment on dry ice. In Cambridge, venous plasma samples were treated with 0.3% tributyl phosphate/1% Tween 80 (both Sigma, Poole, UK) to inactivate encapsulated viruses. Samples were stored at −80°C prior to analysis.

### Serology

SWA-IgE levels and SWA-IgG4 levels in venous plasma samples were measured in duplicate by ELISA. 384-well plates were coated with 8 µg/ml SWA antigen. For IgE, plasma was diluted 1∶20 and SWA-IgE levels detected using anti-human IgE antibody clone G7-26 (BD Pharmingen, San Diego, CA). For IgG4, plasma was diluted 1∶200 and SWA-IgG4 levels detected using anti-human IgG4 antibody clone G17-4 (BD Pharmingen). Assays were developed using OPD substrate (Sigma). SWA-IgE concentrations were extrapolated from standard curves based on a pool of SWA-IgE positive sera from previous studies that had been quantified by the ImmunoCAP assay (Phadia, Uppsala, Sweden). SWA-IgG4 concentrations were extrapolated from purified human IgG4 myeloma (Sigma) derived standard curves.

IL-4, IL-5, IL-13 and eotaxin levels were measured in finger-prick plasma samples by Luminex bead array. Beads were coupled with capture monoclonal Ab (IL-4, IL-5, and IL-13, BD Pharmingen; Eotaxin, R&D Systems, Minneapolis, MN), incubated with 12.5 µl plasma, diluted 1∶8, overnight at 4°C, and levels detected using monoclonal Ab for IL-4, IL-5 and IL-13 (BD Pharmingen), and poly-clonal goat anti-human eotaxin (R&D Systems).

### Analysis of Data

*S. haematobium* infection intensities, SWA-IgE, eosinophil numbers and plasma IL-5, IL-13 and eotaxin levels were log-transformed prior to statistical modeling. Age-profiles of some variables modeled are not linear, so age was divided into an ordinal variable; in models of IL-5 at 24-hr and SWA-IgE and eosinophils at 9-wk post-treatment, age-groups were: 5–6 yr (n = 63), 7–9 yr (n = 59), 10–14 yr (n = 53), 15–23 yr (n = 48), 24–34 yr (n = 65), and 35–40 yr (n = 38). The number of young adults who participated 2-yr post-treatment was insufficient to maintain these age-groups for re-infection models, so age-groups were collapsed to pre-treatment ages of 5–6-yr (n = 34), 7–9 yr (n = 32), 10–14 yr (n = 21), 15–30 yr (n = 33) and 31–40 yr (n = 32). Students t-test of log-transformed variables were used to compare means of two groups, and paired t-tests to compare means of log-transformed longitudinally matched data. Prevalence was compared using Chi-squared tests.

Linear regression models were constructed, except for models of re-infection 2-yr post-treatment, for which logistic regression models were constructed. Village of residence, sex and age group were added *a priori* into initial models. Models were reduced using backwards stepwise regression via the step command which applies Akeike's information criterion (AIC) to test all variables for their requirement in achieving maximum fit of the model. Further removal of variables with a p-value >0.05 was carried out, least significant first; ANOVA was used to compare the final reduced model with that returned by the step function to confirm that removal of these variables did not significantly diminish the fit of the model. The exception to reduction by backward regression was when age-group was removed in models of re-infection to detect any masking of potential immune correlates by this confounder.

## Results

### Demographic Characteristics and Parasitology

The geometric mean age, sex ratios, village of residence and geometric mean pre-treatment *S. haematobium* infection intensity of the initial cohort, and those successfully followed up at 9-wks and 2-yrs post-infection, are shown in table 1. The fishing villages of Kaladangan and Guenidaga had a greater loss to follow-up than Kalabougou (χ^2^ = 38.33, p<0.001). All other variables remained similar amongst the initial cohort and those successfully followed up, including the immune parameters that were used in further analysis (data not shown).

**Table 1:** Baseline *S. haematobium* infection intensity and demographic characteristics of initial and post-treatment follow-up cohorts.

		Baseline	9-wk	2-yr
Age (yrs)		13.8 (12.8, 14.9)	13.4 (12.3, 14.6)	12.8 (11.4, 14.4)
Sex (n (%))	Female	186 (57.1%)	141 (57.1%)	96 (60%)
	Male	140 (42.9%)	104 (42.9%)	64 (40%)
Village (n (%))	Kalabougou	179 (54.9%)	149 (60.8%)	117 (73.1%)
	Kaladangan and Guenidaga	149 (45.1%)	96 (39.2%)	43 (26.9%)
*S. haematobium* (eggs/10 ml)		14.3 (11.5, 17.8)	15.0 (11.7, 19.2)	13.9 (10.2, 18.9)

Geometric mean and 95% confidence interval are shown for age and *S. haematobium* infection intensity.

Pre-treatment *S. haematobium* infection intensities were high, even in the youngest age-group, and peaked at 7–9 yrs of age before characteristically declining in older children and adults. There was no difference in infection intensities between the sexes (males: geometric mean = 17.02 eggs/10 ml (95% CI: 12.17, 23.81); females: geometric mean = 12.55 eggs/10 ml (95% CI: 9.42 16.73), t = −1.36, p = 0.174). There was a village effect on infection. The prevalence in Kalabougou was 74.3%, with a geometric mean infection intensity of 8.03eggs/10 ml (95% CI: 6.13, 10.52). In Guenidaga and Kaladangan combined, the prevalence of *S. haematobium* infection was 91.2%, significantly higher than that in Kalabougou (χ^2^ = 14.35, p<0.001), and the geometric mean infection intensity was also significantly higher (t = −5.057, p<0.001) at 28.89 eggs/10 ml (95% CI: 20.95, 39.83). Treatment successfully cleared all *S. haematobium* infections, except for 30, 6 from Kalabougou, and 24 from Kaladangan and Guenidaga. Infection intensities at 9-wk for these 30 individuals were greatly reduced (geometric mean = 3.89 (95% CI: 2.57, 5.88)). There was no significant difference in pre-treatment prevalence (χ^2^ = 0.6791, p = 0.410), infection intensity (t = 0.4391, p = 0.661) nor in treatment efficacy (χ^2^ = 0.132, p = 0.716) or infection intensities of those whose *S. haematobium* infection was not cleared (t = −1.225, p = 0.233) between the two praziquantel regimes. Nor were there significant differences between the two regimes in any of the immune parameters analysed below (data not shown). Only 35 of the 252 individuals who provided stool samples had detectable *S. mansoni* eggs (P = 13.9%), 33 of whom were from Kaladangan or Guenidaga, and for 3 of whom treatment did not clear infection. No gut nematode eggs were detected.

### Plasma Cytokine Levels 24-hours Post Treatment

**Figure 1:**
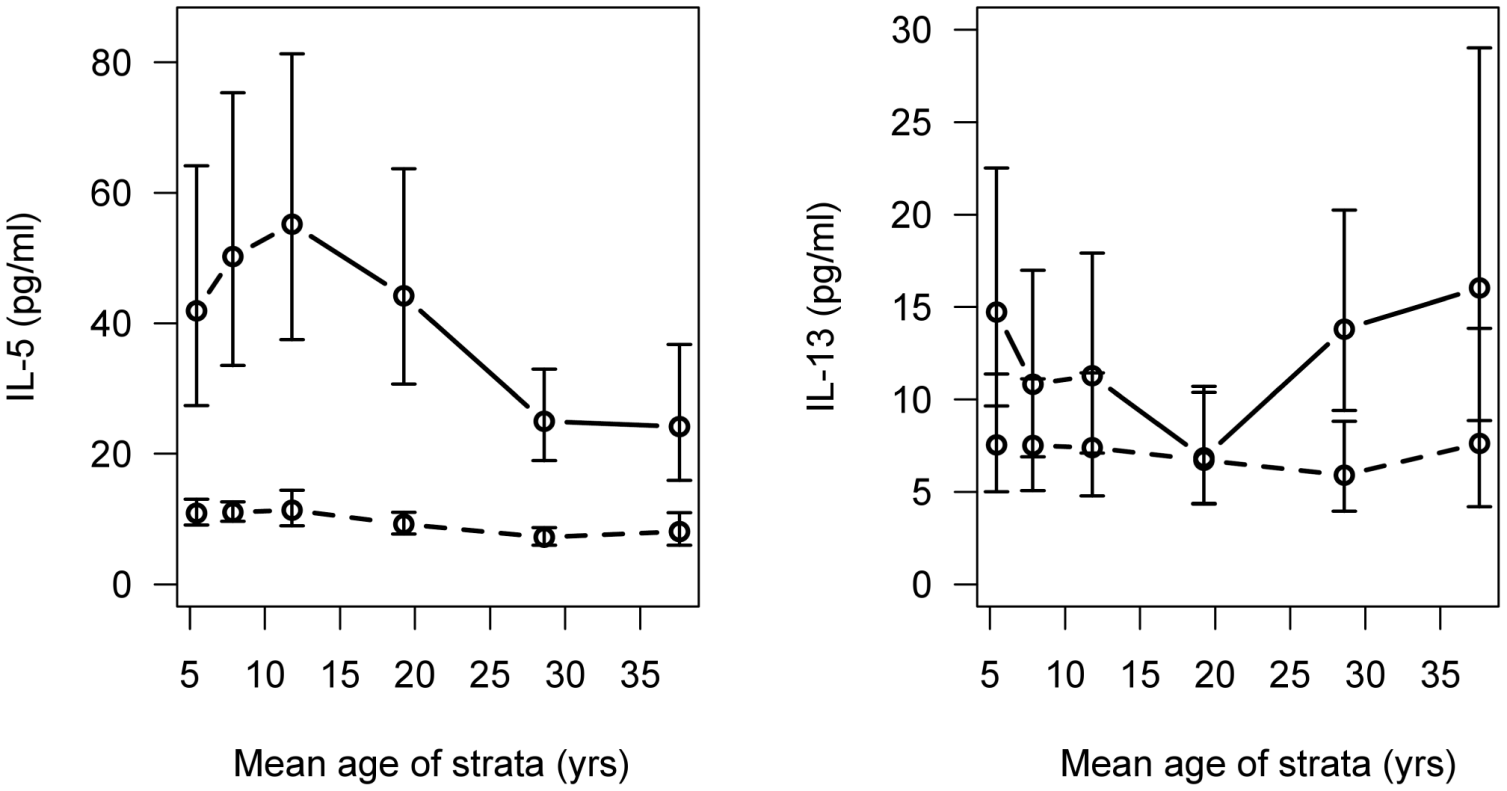
IL-5 and IL-13 plasma levels pre and post-treatment by age. Shown are the geometric mean plasma IL-5 and IL-13 levels and 95% confidence intervals, pre-treatment (dotted line) and 24-hours post-treatment (solid line) by age.

The geometric mean IL-5 level pre-treatment was 9.54 pg/ml (95% CI: 8.80, 10.36). At 24-hr post-treatment the geometric mean IL-5 level was 38.62 pg/ml (95% CI: 33.04, 45.14). This boost in IL-5 levels was significant (t = −19.37, p<0.001) and was in all age-groups (fig. 1). The profile of the IL-5 boost with age (fig. 1) was similar to that of infection intensity with age, however, the peak in the boost occurred slightly later (10 to 14-yr for IL-5 levels, 7 to 9-yr for infection intensity). There was no significant difference in IL-4 levels pre-treatment and 24-hr post-treatment, and they were very low at both time-points (pre-treatment geometric mean = 3.17 pg/ml (95% CI: 2.98, 3.36); 24-hr post-treatment geometric mean = 3.75 pg/ml (95%CI: 3.48, 4.04), t = −0.95, p = 0.340). The pre-treatment geometric mean IL-13 level was 7.04 pg/ml (95% CI: 5.91, 8.37), and was significantly boosted 24 hr post-treatment (t = −5.01, p<0.001), when the geometric mean levels were 11.87 pg/ml (95%CI: 9.90, 14.24). Regression analysis of 24-hr IL-13 levels, controlling for pre-treatment IL-13 levels, showed that the only significant trend with age was due to the failure of the 15–23 yr olds to boost IL-13 24 hr post-treatment (fig. 1, β = −0.73, S.E. = 0.32, p = 0.016).

Linear regression models of 24-hr post-treatment plasma IL-5 levels were constructed, controlling for pre-treatment IL-5. Infection intensity, and the known correlates of immunity, SWA-IgE levels and eosinophil number, were measured pre-treatment, and the 24-hr levels of the eosinophil attractant, eotaxin, was measured as an indicator of its release in response to treatment. These parameters and the *a priori* variables of village group, sex and age-group were entered into the model. In the initial model neither age-group nor *S. haematobium* infection intensity were significant predictors of the boost in IL-5 (table 2). After the removal of age-group from the model during backwards stepwise regression, *S. haematobium* infection intensities were significantly associated with IL-5 levels at 24-hr post-treatment (table 2; reduced model). Pre-treatment levels of SWA-IgE and eosinophil numbers, and the plasma eotaxin levels at 24-hr post-treatment were also significant predictors of the boost in IL-5 (table 2). The model was not improved by the addition of detectable *S. mansoni* infection (data not shown).

**Table 2:** Linear regression model of the boost in levels of plasma IL-5.

	Full model	Reduced model
	β (SE)	β (SE)
Sex	0.201 (0.131)	-
Village-group	0.319 (0.141)*	0.305 (0.128)*
Age (v. 5–6 yrs) 7–9	0.215 (0.213)	-
10–14	0.135 (0.223)	-
15–23	0.091 (0.243)	-
24–34	−0.067 (0.225)	-
35–40	−0.355 (0.252)	-
log infection intensity	0.053 (0.037)	0.071 (0.035)*
log pre-treatment SWA-IgE	0.191 (0.050)***	0.189 (0.047)***
log pre-treatment eos. no	0.397 (0.094)***	0.418 (0.092)***
log 24 hr eotaxin	0.341 (0.071)***	0.333 (0.071)***

* p<0.05,

*** p<0.001.

### Eosinophil Number 9-weeks Post-treatment

Pre-treatment circulating eosinophil number was 4.53×10^5^ cells/ml (95% CI: 4.11×10^5^, 4.98×10^5^). Nine-wk post-treatment the geometric mean number of circulating eosinophils had increased to 5.27×10^5^ cells/ml (95% CI: 4.81×10^5^, 5.76×10^5^). This increase in circulating eosinophil number was significant (t = −3.11, p<0.001). Models of eosinophil number at 9-wk post-treatment were constructed, controlling for pre-treatment eosinophil number. The same variables as those modeled for the IL-5 boost: i.e. sex, village-group and age-group, pre-treatment infection intensities, SWA-IgE levels and 24-hr post-treatment eotaxin, were entered, along with IL-5 levels 24-hr post-treatment. IL-5 levels 24-hr post-treatment were significantly associated with increased eosinophil number at 9-wk post-treatment (table 3), as were pre-treatment infection intensities. Pre-treatment SWA-IgE levels, sex, age-group, village-group and eotaxin levels at 24-hr post-treatment, did not contribute to the model and were removed (table 3, reduced model). This did not significantly diminish the fit of the model.

**Table 3:** Linear regression models of the boost in eosinophil number at 9 weeks post-treatment.

	Full model	Reduced model
	β (SE)	β (SE)
Sex	0.113 (0.079)	-
Village-group	0.132 (0.085)	-
Age (v. 5–6 yrs) 7–9	0.027 (0123)	-
10–14	−0.016 (0.133)	-
15–23	−0.035 (0.146)	-
24–34	0.010 (0.132)	-
35–40	−0.170 (0.152)	-
log infection intensity	0.054 (0.022)*	0.061 (0.020)**
log pre-treatment SWA-IgE	0.007 (0.029)	-
log 24 hr eotaxin	−0.056 (0.045)	-
log 24 hr IL-5	0.115 (0.032)***	0.121 (0.028)***

* p<0.05,

** p<0.01,

*** p<0.001.

### SWA-IgE Antibody Levels 9-weeks Post-treatment

**Figure 2:**
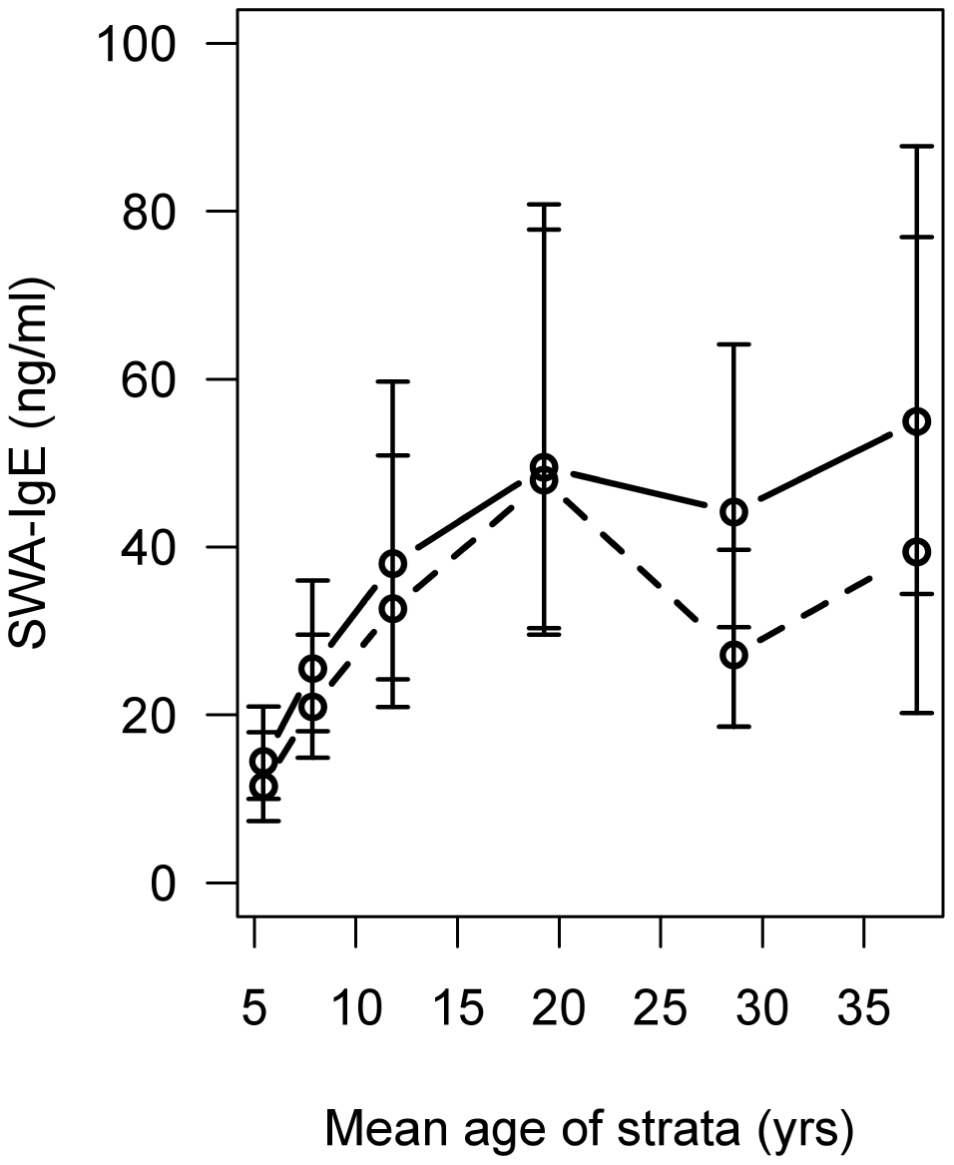
SWA-IgE levels pre and post-treatment by age. Shown are the geometric mean SWA-IgE levels and 95% confidence intervals, pre-treatment (dotted line) and 9-weeks post-treatment (solid line) by age.

SWA-IgE levels pre-treatment were 25.07 ng/ml (95% CI: 20.87, 30.11) and significantly increased in the cohort as a whole at 9-wk post-treatment (t = −3.9982, p<0.001) when the geometric mean level was 32.12 ng/ml (95% CI: 27.07, 38.12). Stratifying the data by age-group, a negligible increase was observed in the younger age-groups, but the increase was prominent in the oldest age-groups (fig. 2). This appears to be due to a pre-treatment decrease in SWA-IgE in these oldest age-groups compared to the 15–23 yr olds, that was no longer apparent post-treatment, when SWA-IgE reached a plateau from 15–25 yrs of age.

Linear regression models of 9-wk post-treatment SWA-IgE, controlled for pre-treatment levels, were constructed. The full model indicated that age-group and 24-hr post-treatment IL-5 levels were significant predictors of 9-wk post-treatment SWA-IgE. None of the other predictors – sex, village-group, pre-treatment infection intensities, eosinophil number and 24-hr post-treatment eotaxin - significantly contributed to the model (table 4), and their removal did not significantly diminish the fit of the model.

**Table 4:** Linear regression models of boost in SWA-IgE at 9 weeks post-treatment.

	Full model	Reduced model
	β (SE)	β (SE)
Sex	−0.174 (0.115)	-
Village-group	−0.077 (0.125)	-
Age (v. 5–6 yrs) 7–9	0.139 (0.179)	0.162 (0.175)
10–14	0.207 (0.193)	0.249 (0.189)
15–23	0.179 (0.212)	0.245 (0.201)
24–34	0.508 (0.192)**	0.608 (0.180)**
35–40	0.541 (0.225)*	0.593 (0.220)**
log infection intensity	−0.052 (0.032)	-
log pre-treatment eos no.	0.036 (0.082)	-
log 24 hr eotaxin	−0.020 (0.065)	-
log 24 hr IL-5	0.112 (0.047)*	0.085 (0.040)*

* p<0.05,

** p<0.01.

When 24-hr post-treatment IL-13 levels replaced 24-hr post-treatment IL-5 levels in the model, IL-13 was not found to be significantly associated with 9-wk post-treatment SWA-IgE levels. Nor did the addition of IL-13 to the model containing 24-hr post-treatment IL-5 levels, alter the significance of the relationship between IL-5 and SWA-IgE (data not shown). Due to the very low levels measured, and the failure to detect a boost in IL-4 at 24-hr post-treatment, this cytokine was not analysed.

SWA-IgG4 levels increased from a pre-treatment geometric mean of 20.74 µg/ml (95% CI: 16.00, 26.88) to a 9-wk post-treatment geometric mean of 43.23 µg/ml (95% CI: 32.06, 58.31). This increase was significant (t = −8.467, p<0.001). In linear regression models of 9-wk post-treatment SWA-IgG4, controlling for pre-treatment levels, sex, village group, age-group, pre-treatment SWA-IgE, pre-treatment eosinophil number, 24-hr eotaxin and 24-hr IL-5 were not significant (data not shown).

### Re-infection 2-years Post-treatment

Of the 152 individuals followed-up at 2-yr post-treatment, who also had no detectable eggs at 9-wk post-treatment, 99 (65.13%) had been re-infected. A logistic regression model of yes/no re-infected was constructed. Individuals who were egg positive at 9-wk post-treatment were not included in this model. Similar to pre-treatment infection, there was a village effect on re-infection, and older age-groups had significantly reduced odds of being re-infected than the youngest age-group. Neither IL-5 levels 24-hr post-treatment nor SWA-IgE levels 9-wk post-treatment were significant predictors of re-infection status (table 5, model 1). As age is closely related to SWA-IgE levels, and is a known confounder of the relationship between post-treatment SWA-IgE levels and re-infection, the model was re-fitted with age-group removed. When age-group was removed from the model, SWA-IgE levels 9-wk post-treatment were negatively associated with re-infection, but IL-5 levels 24-hr post-treatment were still not significant (table 5, model 2). Eosinophil count at 9-wk post-treatment was positively associated with re-infection in this second model. SWA-IgG4 was added to these models as SWA-IgG4 has previously been reported to be associated with susceptibility to re-infection. It was not significantly associated with re-infection status in either the model with age-group or the model without age-group (table 5). The model that included age-group had a better fit (AIC = 134.87), than the model that did not include age-group (AIC = 171.17).

**Table 5:** Logistic regression models of re-infection status.

	Model 1 (with age-group)	Model 2 (without age-group)
	Odds Ratio (95% CI)	Odds Ratio (95% CI)
Sex	0.830 (0.30, 2.26)	1.401 (0.63, 3.23)
Village-group	25.374 (6.74, 123.16)***	6.748 (2.19, 26.39)**
Age (v. 5–6 yrs) 7–9	1.997 (0.39, 11.90)	-
10–14	0.360 (0.07, 1.80)	-
15–30	0.045 (0.01, 0.21)***	-
31–40	0.030 (0.01, 0.15)***	-
log 24 hr IL-5	0.657 (0.41, 1.03)	1.05 (0.75, 1.48)
log 9-week SWA-IgE	0.985 (0.68, 1.44)	0.706 (0.52, 0.95)*
log 9-week eos. no.	2.337 (1.00, 5.86)	2.469 (1.25, 5.20)*
Log 9-week SWA-IgG4	1.056 (0.82, 1.36)	0.887 (0.72, 1.08)

* p<0.05,

** p<0.01,

*** p<0.001.

## Discussion

High levels of IgE are characteristic of helminth infections and of allergy. For some helminth infections, such as schistosomiasis, high levels of circulating parasite-specific IgE are associated with partial immunity to re-infection after chemotherapeutic cure. Control of IgE production is relatively poorly understood, with little known about early cellular and cytokine responses after antigen/allergen exposure and how they relate to later IgE production. Increased IgE levels to worm derived Ag after treatment, in combination with the re-infection study design used in the current study, allows the examination of the early post-treatment responses and their relationship with the later protective IgE response.

Previously, in a small cohort of *S. mansoni* infected Ugandan fishermen, we showed that plasma IL-5 levels are boosted 24-hr post-treatment [17]. In the current study, conducted in an area where high transmission of *S. haematobium* occurs, praziquantel-induced death of *S. haematobium* adult worms also resulted in boosted levels of plasma IL-5. *S. haematobium* adult worms live in the bladder plexus, unlike *S. mansoni* worms, which reside in the intestinal mesenteric veins. The treatment induced circulating IL-5 boost is therefore neither schistosome species-specific, nor dependent on site of worm death. A similar boost in circulating IL-5 has been observed in seasonal allergic rhinitis patients 24-hr after nasal allergen challenge. In that study the increase in plasma IL-5 significantly correlated with concurrent increases in eosinophil cationic protein in sputum [23]. In *S. mansoni* infected fishermen a substantial fall in the numbers of circulating eosinophil, suggesting a rapid migration from the circulation into the tissues, coincided with the 24-hr post-treatment increase in circulating IL-5 [17]. In the current study, data on 24-hr post-treatment eosinophil numbers was not available. However, when the boost in plasma IL-5 at 24-hr post-treatment was modeled, pre-treatment eosinophil number and 24-hr post-treatment levels of plasma eotaxin, a chemokine involved in attraction and migration of eosinophils into tissue [24], were significant. The 24-hr eotaxin levels are likely to indicate the immediate release of this chemokine in response to treatment.

The significance of pre-treatment eosinophil count in the production of IL-5 does not directly implicate them as the source of the IL-5, although they do pre-package IL-5 in their granules [25]. Eosinophils can skew and maintain immune responses via selective release of cytokines [26], [27] but they can also up-regulate co-stimulatory molecules and present antigen [28], [29]. The eosinophils could therefore be responding to early signals to migrate to sites of inflammation, where they drive IL-5 production by T cells. 24-hr post-treatment whole blood cultures release less IL-5 in response to SWA-stimulation than pre-treatment cultures, a responsiveness that is recovered 3-weeks post treatment [17]. A similar inverse relationship between decreased PBMC IL-5 responsiveness and increased plasma IL-5 is also seen early after treatment for lymphatic filariasis [30], suggesting that the lymphocytes capable of producing IL-5 have left the circulation.

As measured in the current study, 24-hr post-treatment plasma IL-5 is part of the in vivo response to challenge as the adult worms' integrity is disrupted in the blood stream, exposing normally cryptic antigen [14]. The association between *S. haematobium* infection intensities, after the removal of the confounder age, and the boost in plasma IL-5 may indicate that the magnitude of the boost in plasma IL-5 levels is dependent on the dose of antigens that the individual is exposed to upon treatment. The plasma IL-5 boost was also SWA-IgE dependent. The dependence on SWA-IgE, a response that increases with age in populations living in schistosomiasis endemic areas [8], [9], [10], may explain why the peak boost in IL-5 occurred in a slightly older age-group than the peak in infection intensities.

Higher circulating SWA-IgE is likely a proxy of increased SWA-specific arming of IgE-effector cells, such as mast cells, which are present in both the gut mucosa and the bladder wall. Mast cells initiate eosinophil migration to the tissue after treatment for *Onchocerca volvulus*, with elevated levels of plasma tryptase, indicative of mast cell degranulation, preceding a decrease in circulating and an increase in skin eosinophil numbers [31]. We have previously observed a small elevation in mast-cell tryptase levels in the plasma at 24-hr post-treatment for *S. mansoni* [32], indicating that a similar mechanism may occur after treatment for schistosomiasis.

IL-5 induces eosinophil maturation and release from the bone marrow [33] and IL-5 levels at 24-hr post-treatment were associated with an increase in eosinophil number at 9-wk post-treatment. A similar burst in IL-5 24–48-hr after treatment, followed by an increase in circulating eosinophil number at 3-days to 2-wk post-treatment, has been seen for the micro-filarial nematodes *O. volvulus* and *Wucheria bancrofti*. For both infections, the time to peak IL-5 levels was significantly associated with the time to peak eosinophil number [30], [34], [35]. As the treatment of schistosomiasis has been shown to induce an increase in eosinophil number, which peaks between 2 and 4-wk post-treatment [16], [17], those individuals who still have elevated circulating eosinophils may have a delayed response. The positive relationship between eosinophil number at 9-wk and re-infection 2-yr later, one that appears surprising in the context of the literature showing pre-treatment eosinophil numbers to be associated with resistance [5], [6], could therefore be due to the study time-point.

Pre-treatment in vitro IL-5 responses specific to SWA are associated with protective SWA-IgE responses in *S. mansoni* infection [22]. Here, plasma IL-5 levels 24-hr post-treatment was the only variable, other than age, that was associated with the increase in SWA-IgE levels at 9-wk post-treatment, so the in vivo, as well as in vitro, IL-5 response is associated with the production of protective SWA-IgE. Class switching of B cells to IgE production is a process predominantly under the control of other Th2 cytokines, IL-4 and IL-13 [36], [37], [38]. As very low levels of IL-4 were measured and no boost was detected, and IL-13 levels at 24-hr post-treatment were not significantly associated with SWA-IgE, the role of these cytokines is likely to be downstream of the IL-5 boost. The observed relationship between 24-hr post-treatment plasma IL-5 levels and 9-wk SWA-IgE could be an indirect relationship, with the IL-5 boost being a proxy of increased Th2 reactivity. An increase in Th2 responsiveness does occur with treatment of schistosomiasis with greater IL-4 and IL-13 in vitro responses to SWA being measured 7-wk, and beyond, post-treatment [20], [39].

SWA-IgE at 9-wk post-treatment was negatively associated with the re-infection that took place over the next 2-yr, but only after the removal of age. SWA-IgE levels at 9-wk post-treatment increased to a plateau with age (but not the pre-treatment levels which declined in the oldest age-groups); due to the close relationship of post-treatment SWA-IgE levels with age, it was not possible to distinguish their relative contribution to re-infection. The improved model fit when age was included indicates that SWA-IgE levels, although likely to be one of the key mediators, are not the only age-related correlate of immunity. However, neither the 24-hr post-treatment IL-5 levels nor the SWA-IgG4 levels at 9-wk post-treatment, an antibody isotype reported to be associated with susceptibility to re-infection [2], [40], were significantly associated with re-infection in this study. Other age-related immune correlates not measured in the present study, could include an increase in CD23 expression by B cells [41]. The expression of CD23, the low affinity IgE-receptor, has been hypothesized to increase specific antigen capture and subsequent presentation to B cells, augmenting IgE production in schistosomiasis [42].

Here we have shown a boost in plasma IL-5 levels 24-hr after human *S. haematobium* infections are treated with praziquantel, and that this is dependent on both the intensity of the pre-treatment infection (or antigen dose the individual is exposed to by treatment) and pre-treatment levels of SWA-IgE. Eosinophils are implicated in this plasma IL-5 boost, as their pre-treatment levels, and the 24-hr post-treatment levels of the eosinophil chemo-attractant eotaxin, are also significantly associated with the IL-5 boost. The IL-5 in turn, is related to 9-wk post-treatment eosinophil number and elevated SWA-IgE levels. SWA-IgE was, in line with previous studies, shown to be associated with re-infection immunity. The observation that an IL-5 boost also occurs in allergic rhinitis patients 24-hr after allergen exposure, and after treatment for micro-filarial infection, indicates that the results presented here may also have relevance to the increased IgE levels observed in patients suffering from seasonal allergy and in immunity to other helminths.

## Supporting Information

Table S1 **STROBE Checklist.**
(DOC)
